# Comprehensive Atlas of Circulating Rare Cells Detected by SE-iFISH and Image Scanning Platform in Patients With Various Diseases

**DOI:** 10.3389/fonc.2022.821454

**Published:** 2022-03-02

**Authors:** Binjie Hu, Yanping Gong, Yulan Wang, Jianzhu Xie, Jin Cheng, Qian Huang

**Affiliations:** Molecular Diagnostics Laboratory of Cancer Center, Shanghai General Hospital, Shanghai Jiao Tong University School of Medicine, Shanghai, China

**Keywords:** circulating rare cells, SE-iFISH, image scanning, comprehensive atlas, cellular biomarkers, double probes, various diseases

## Abstract

**Objective:**

Circulating rare cells (CRCs) are known as a crucial nucleated cellular response to pathological conditions, yet the landscape of cell types across a wide variety of diseases lacks comprehensive understanding. This study aimed at detecting and presenting a full spectrum of highly heterogeneous CRCs in clinical practice and further explored the characterization of CRC subtypes in distinct biomarker combinations and aneuploid chromosomes among various disease groups.

**Methods:**

Peripheral blood was obtained from 2,360 patients with different cancers and non-neoplastic diseases. CRC capture and identification were accomplished using a novel platform integrating subtraction enrichment and immunostaining-fluorescence *in situ* hybridization (SE-iFISH) strategy with a high-throughput automated image scanning system, on which hemocyte, tumor, epithelial, endothelial, mesenchymal, and stemness biomarkers were immunostained and displayed simultaneously. Double chromosome enumeration probe (CEP8 and CEP12) co-detection was performed on isolated CRCs from an extended trial for two chromosome ploidy patterns.

**Results:**

A comprehensive atlas categorizing the diverse CRCs into 71 subtypes outlining was mapped out. The presence of epithelial–mesenchymal transition (EMT) or endothelial–mesenchymal transition (EndoMT), the cells with progenitor property, hematologic CRCs expressing multiple biomarkers, CRCs at “naked nuclei” status, and the rarely reported aneuploid mesenchymal epithelial–endothelial fusion cluster were described. Circulating tumor cells (CTCs) were detected in 2,157 (91.4%) patients; the total numbers of CTCs and circulating tumor-derived endothelial cells (CTECs) were relatively higher in several digestive system cancer types and non-neoplastic infectious diseases (*p* < 0.05). Co-detection combining CEP8 and CEP12 showed a higher diagnostic specificity on account of 57.27% false negativity of CRC detection through a single probe of CEP8.

**Conclusions:**

The alternative biomarkers and chromosomes to be targeted by SE-iFISH and the image scanning platform, along with the comprehensive atlas, offer insight into the heterogeneity of CRCs and reveal potential contributions to specific disease diagnosis and therapeutic target cell discovery.

## Introduction

In today’s comprehensive oncology era, clarification of abnormal cell landscapes of individual patients’ peripheral circulation is crucial to early diagnosis, therapeutic guidance, prognostic monitoring, and therefore alertness for the emergence of treatment resistance, tumor metastasis, and disease relapse. Considering the challenges of biopsy acquisition, the tumor evolution during disease progression, and especially the temporal and spatial heterogeneity of the tumor cells (1), there is a strong need for an accessible method implying minimally invasive procedures and allowing real-time monitoring of patients’ tumor cell alterations (2). Liquid biopsy, defined as the capture of disease-related cells shed from solid tumor or molecules during the apoptosis and necrosis of tumor cells in a fluid sample (3), has been extensively studied and widely used in clinical practice. The approach is composed of different biological matrices such as circulating tumor cells (CTCs), circulating tumor deoxyribonucleic acids (ctDNA), circulating cell-free deoxyribonucleic acids (cfDNA), microRNA (miRNA), and tumor-derived extracellular vesicles (2, 4). Among them, the measurement and analysis of CTCs are of great importance to access the macroscopical information of tumor cells and have raised considerable attention.

In the case of blood circulation, CTCs are a population of cells that accompany primary tumor cell invasion into the bloodstream, escape anoikis, evade destruction by the immune system, survive and settle in secondary or remote organs (5), and therefore play a critical role in cancer relapse and metastasis and the management of advanced diseases. A variety of cell-based liquid biopsies have been explored to find the appropriate CTC enrichment and identification technologies, and most of the current detection strategies rely on physical cellular properties or cell surface molecules for isolation, and immunostaining of cellular proteins for identification (6).

To obtain sufficient numbers of CTCs for meaningful follow-up studies, alternative cell isolating methods, mainly consisting of cell filtration, positive selection, and negative enrichment based on different principles (7), were developed to date. Cell size-based filtration, such as ISET (isolation by size of epithelial tumor cells) (8), relies on the assumption that CTCs are derived from the epithelium which are larger than white blood cells (WBCs) and clusters of CTCs or those circulating tumor microemboli (CTM) would be rapidly isolated. Nevertheless, recent studies have proved the existence and clinical effects of CTCs with the size similar to or smaller than WBCs (9, 10), one inherent limitation of such technique that loss of a significant amount of small CRCs should not be neglected (11). Another kind of widely used technology is based on positive selection, in which the anti-epithelial cell adhesion molecule (EpCAM) and/or cytokeratin (CK) coated on alternative carriers recognize and capture the CTCs, including the FDA-approved CellSearch^®^ system (12, 13), flow cytometers (14), CTC-chip (15), and fiber-optic array scanning technology (FAST) (16). The availability of highly specific antibodies against tumor surface antigens remarkably improved the specificity and the success rates of isolating CTCs. However, emerging evidence has revealed highly dynamic localization and expression of targeted anchor proteins on tumor cells among different tissues or even within the same sample (17, 18). In addition, it is also reported that intracellular signaling pathways of cancer cells could be activated by cross-linking of cell surface molecules (such as EpCAM) following antibody binding (19), perturbing subsequent analyses. More importantly, a distinct phenotype of mesenchymal CTCs, along with aneuploid circulating tumor-derived endothelial cells (CTECs) that possess the dual properties of cancerous malignancy and endothelial vascularization ability, has also been discovered in the tumor neovascularization process, which involves both epithelial–mesenchymal transition (EMT) and endothelial–mesenchymal transition (EndoMT) (20–22), while the expression of EpCAM and/or CK in CTCs might be downregulated or absent during the process of EMT (23), potentially resulting in significant false-negative detection of CTCs by a single tumor marker. Contrary to the positive selection, negative enrichment applies both hypotonic lysis and anti-CD45 antibody to remove red blood cells (RBCs) and WBCs, respectively (24), and therefore enriches CTCs in the eluted cell suspension. This method exposes fragile CTCs to deleterious hypotonic damage and mechanical stresses (25), consequently interfering with the detection efficiency. Accordingly, a novel CTC detection technology SE-iFISH, which combines *in situ* phenotypic identification of more than one biomarker and karyotypic characterization of chromosome ploidy in CTCs enriched from patients’ peripheral blood, using subtraction enrichment (SE), independently of cell size, cluster, CTM, or surface anchor protein expression, has been developed (7, 26).

Distinguished from the conventional negative enrichment, the SE strategy effectively and efficiently removes RBCs *via* centrifugation other than applying hypotonic damage (27). Moreover, a special coating of the immuno-magnetic beads conjugated to antibodies unique for multiple surface antigens of WBC ensures minimum non-specific binding of other nucleated cells to the magnetic particles and maximal removal of WBCs (approx. 4–5 logs) (7). About CTC identification, aneuploidy of chromosomes identified by chromosome enumeration probe (CEP) in neoplastic cells has been observed in various types of cancer (28, 29), while immunostained proteins are proved to be unrestricted to either intracellular or extracellular antigenic epitopes of nuclear, cytosolic, and membrane-associated tumor biomarkers as well as additional markers specifying the epithelial, endothelial, mesenchymal, hematopoietic, or stem cell nature (30). Therefore, the established immunostaining-FISH (iFISH) approach provided numerous choices of the desired biomarkers to be targeted and any of the chromosomes to be investigated.

With the clinical application of SE-iFISH, there are an increasing number of accidental findings and occasional investigations on various phenotypes of CTCs other than conventional tumor cell types. Stefan Schreier et al. have raised a new comprehension of circulating rare cells (CRCs) that CRCs may be coarsely defined as nucleated cellular events with different differentiation potential in peripheral blood reflecting some degree of the physiological condition of the tissue of origin, and share the complementary association with certain diseases (31). However, with greater diversity of possible biomarkers, correct phenotyping of rare cells detected in clinical practice has not been explored in previous investigations.

In awareness of more CRC types that extend beyond the current spectrum, this study intends to expand the varieties of diseases and the number of clinical samples as much as possible. Analysis of the expression of multiple biomarkers, preliminary research on the quantification of aneuploid CTCs/CTECs in various disease types, and co-detection of aneuploidy of double centromere probes of chromosomes 8 and 12 were also carried out. Here, 71 subtypes of CRCs among 31 groups of disease types were classified by cell size, biomarkers, and chromosome ploidy, and a comprehensive atlas was mapped out for the first time. The results would set the foundation for further clinical assessment of therapy effectiveness and provide new ideas of tumor cell heterogeneity in the tumor microenvironment as well as peripheral circulation of carcinoma individuals.

## Materials and Methods

### Patients and Samples

This is a retrospective study conducted on patients who had taken the CRC detection strategy from November 2016 to January 2021 in Shanghai General Hospital (Shanghai, China). A total of 3,476 peripheral blood samples were collected from 2,360 patients with various confirmed cancers, diagnosed non-neoplastic diseases, and undetermined pulmonary nodules. The pathological diagnosis, the TNM staging data of cancers, and the treatment histories were all documented in detail. The patients with diagnosed cancers under regular follow-up of tumor recurrence and metastasis; the patients under tumor treatment cycles including chemotherapy, radiotherapy, interventional therapy, targeted drug therapy, or immunotherapy for treatment baseline assessment, tumor progression monitoring in the therapeutic process, and curative effect observation; and the patients who suffered from surgery with suspected malignancy under imaging indication for dynamic observation in a perioperative period met our initial inclusion criteria. There is no cancer type and disease stage restriction. The blood samples were drawn at the regular follow-up examination, and multiple test samples from the same patients were collected before, during, and upon completion of every cancer treatment cycles, respectively, or before and after surgery if the operation was necessarily taken. Blood samples were stored at room temperature away from light, and all experiments were performed within 24 h after sample collection.

### Subtraction Enrichment of CRCs

The subtraction enrichment experiment was performed at room temperature according to the updated kit instructions (Cytelligen, San Diego, CA, USA) with certain published modifications (26). 6 ml of patient peripheral blood was collected into a tube containing acid citrate dextrose (ACD) anti-coagulant solution (Becton-Dickinson, Franklin Lake, NJ, USA) in the routine blood sampling, or after discarding the first 2 ml of blood to avoid epithelial cell contamination. The tube was immediately inverted to mix the sample and then centrifuged for 15 min (300 × g), the supernatant plasma was discarded, and the blood cell pellets were diluted with approximately 3 ml of resuspension buffer, mixed gently, and loaded to the upper layer of 3 ml of the sample density separation matrix in a 50-ml tube, subsequently centrifuged at 350 × g for 6 min. The sample was divided into three layers after centrifugation, and on top of the RBC layer, the upper buffy coat solution containing nuclear cells was extracted into a new 50-ml tube and incubated on a gentle shaker for 20 min after adding to 300 μl of washed immuno-magnetic beads, which conjugated with a cocktail of anti-leukocyte monoclonal antibodies. Finally, WBCs bound to beads were removed by passing the mixed sample through a magnetic frame (Promega, Madison, WI), the bead-free solution was then transferred into a new centrifuge tube and thoroughly washed with resuspension buffer two times at 500 × g for 5 min, and non-hematopoietic circulating cells were enriched in the remaining liquid.

### Immunostaining-Fluorescence *In Situ* Hybridization

100 μl of the enriched cell suspension was added to a 2-μl antigen repair buffer and shaken gently for mixing. After standing for 10 min away from light, the cell pellet of each blood sample was subjected to the mixed immunostaining solution with 200 μl blood cell analysis diluent, 1 μl Alexa Fluor 594-conjugated monoclonal anti-CD45 solution, 1 μl Cyanine 5-conjugated monoclonal anti-CD31 solution, 1 μl Cyanine 7-conjugated monoclonal anti-Vimentin solution, and 1 μl Alexa Fluor 488-conjugated monoclonal anti-EpCAM/CK18/PD-L1/AFP/HER2/CA19-9/CD133 solution for six-channel iFISH. Due to the fluorescence channel limit, we used a fixed immunostaining panel of the CD45/CD31/Vimentin/tumor marker and selected the eligible tumor marker based on the source of tumor for each sample. The same individual patient could be sampled more than once at one test timepoint for special tumor markers if necessary. Then the mixture was incubated for 20 min in the dark and washed with resuspension buffer by centrifuging at 500 × g for 5 min to remove excess antibodies. The left 100-μl cell suspension after discarding the supernatant was fixed with an equal volume of fixative and loaded onto the formatted cell slide (Cytelligen). The slide was dried in the air-dried oven at 34°C overnight. The cells on the slide were fixed again the next day followed by hybridization (denaturation at 76°C for 10 min and hybridization at 37°C for 3 h) using the S500-24 Statspin ThermoBrite Slide Hybridization/Denaturation System (Abbott Molecular, Des Plaines, IL, USA) with chromosome enumeration probe 8 (CEP8, Vysis, Abbott Laboratories, Abbott Park II, USA) for single-probe detection, or CEP8 and CEP12 (Alexa Fluor 488-conjugated) for double-probe detection, respectively. After the hybridization was completed, the sample area was washed again to reduce non-specific staining and dried with a hairdryer. In the end, 10 μl DAPI dye solution (Vector Laboratories, Burlington, CA, USA) for nucleus staining was added to the cell area and the sample slide was immediately observed under a fluorescence microscope or preserved at 4°C in the dark within 3 days.

### High-Throughput Automated Image Scanning and Analysis of CRCs

**Figure 1:**
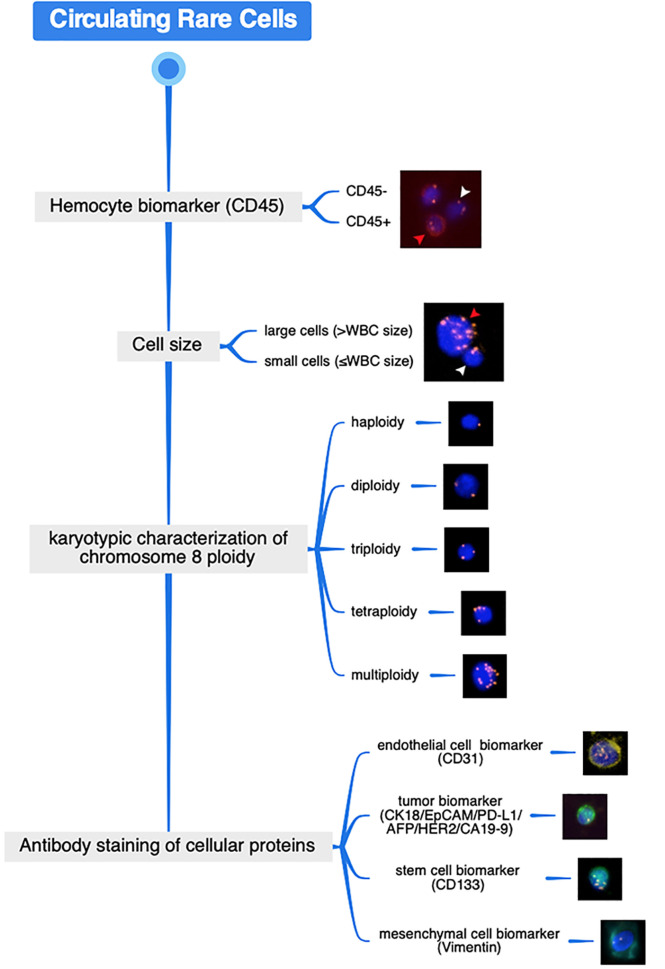
Schematic diagram of CRC analysis procedure through SE-iFISH and image scanning platform and the representative cellular images. Distinguished from WBC by hemocyte biomarker (red arrow represents WBC (CD45^+^) whereas white arrow represents the CD45^-^ non-hematologic cell) and cell size discrimination (red arrow represents the large cell whereas white arrow represents the small cell), karyotypic FISH carried out using an enumeration probe of chromosome 8 and *in situ* phenotypic immunostaining of multiple biomarker proteins are successively performed on the identical enriched target cell.

The image acquisition and the characteristic analysis of CRCs on the slide were performed using a high-throughput automated Metafer-iFISH image scanning system (Carl Zeiss, Oberkochen, Germany; MetaSystems, Altlussheim, Germany; and Cytelligen, San Diego, CA, USA). To acquire the entire fluorescence signal of each multicolor channel, every sample slide was subjected to automated full X–Y plane scanning with cross Z-sectioning of all cells performed at 1-mm steps of depth. ×10 magnification (Zeiss Plan-Apochromat 10x/0.45 M27) was used to scan the sample slide, and exposure times were auto-selected. All images were independently reviewed by two skilled investigators. The analysis procedure of the scanning images as well as the representative cells were schematically depicted and simultaneously shown (**Figure 1**). CRC classification was performed by the sequence of hemocyte biomarker (CD45), cell size, karyotypic characterization of chromosome 8 ploidy, and antibody staining of cellular proteins including endothelial cell biomarker (CD31), epithelial/tumor biomarker (EpCAM/CK18/PD-L1/AFP/HER2/CA19-9), stem cell biomarker (CD133), and mesenchymal cell biomarker (Vimentin). In conventional sense, typical CTCs are identified as CD45^-^/DAPI^+^/CD31^-^/Vimentin^-^/tumor biomarker^+^ phenotype or CD45^-^/DAPI^+^/CD31^-^/Vimentin^-^/tumor biomarker^-^ with aneuploid chromosome 8, while CTECs are identified as CD45^-^/DAPI^+^/CD31^+^/Vimentin^-^/tumor biomarker^+/-^ with aneuploid chromosome 8.

### Statistical Analysis

Statistical analysis of the data was carried out with standard software (IBM SPSS Statistics 26.0, USA); landscapes based on the results were formulated by using the package of *ggplot2* in R version 4.1.0 (http://www.r-projrct.org/). The Kruskal–Wallis *H*-test was used for a statistical comparison of total numbers of CTCs and CTECs among different disease groups. A two-tailed *t-test* was used to compare chromosome aneuploidy detection efficiency between two chromosome enumeration probes (CEP8 and CEP12). All p values were two-sided, and *p* < 0.05 was considered statistically significant.

## Results

### Patient Characteristics and Total CTC Detection

We broadly applied SE-iFISH and image scanning platform to detect CRCs in peripheral blood of a total of 1,968 cancer patients from 25 primary tumor locations, respectively, 92 cancer patients with multiple tumor primary, and 11 patients with cancers of unknown primary. In addition, 106 individuals with undetermined pulmonary nodules and 183 non-neoplastic patients were also subjected to the study, including 20 patients with precancerous lesions, 111 patients with benign lesions, and 52 patients with infectious diseases. The non-neoplastic diseases include diverse types of clinical entities, and over 30 types of diseases were represented under the “benign diseases” group, although some disease types were represented by only a small number of samples (**Tables S1–S3**). The overall percentage of CTC positive (CTC number > 0) patients was 91.4%; detailed information is summarized in **Table 1**.

**Table 1:** Clinical characteristics and overall CTC-positive rate of enrolled patients in different disease types.

Disease type	Total number, *n*	Gender, *n (%)*		Age, *median (range)*	Positive of CTCs (%)
		Male	Female		
Lung cancer	373	214	159	64 (22–86)	350 (93.8%)
Colorectal cancer	310	175	135	64 (29–86)	295 (95.2%)
Cervical and uterus cancer	223	0	223	51 (26–85)	203 (91.0%)
Gastric cancer	179	115	64	56 (35–73)	166 (92.7%)
Nasopharynx cancer	131	102	29	57 (29–83)	101 (77.1%)
Liver cancer	116	97	19	59 (34–89)	103 (88.8%)
Breast cancer	104	1	103	54 (26–80)	95 (91.3%)
Laryngeal cancer	89	79	10	64.5 (38–87)	74 (83.1%)
Pancreatic cancer	76	48	28	63 (38–82)	68 (89.5%)
Esophagus cancer	71	58	13	65 (38–85)	69 (97.2%)
Ovarian cancer	54	0	54	57.5 (27–90)	49 (90.7%)
Glioma	33	21	12	49 (10–72)	31 (93.9%)
Cholangiocarcinoma	31	15	16	64 (42–94)	26 (83.9%)
Prostatic cancer	29	29	0	69 (50–89)	27 (93.1%)
Bladder cancer	25	18	7	68 (36–86)	25 (100.0%)
Lymphoma	20	13	7	66 (27–84)	19 (95.0%)
Maxillofacial tumors	19	15	4	67 (44–79)	15 (78.9%)
Salivary gland tumors	14	10	4	65.5 (16–83)	13 (92.9%)
Sarcoma (all)	14	7	7	48 (15–80)	9 (64.3%)
Thymus and mediastinum tumors	13	10	3	59 (38–73)	13 (100.0%)
Renal cancer	12	11	1	65 (57–73)	10 (83.3%)
Ampulla cancer	12	5	7	62.5 (41–76)	12 (100%)
Skin cancer	8	5	3	65 (49–73)	7 (87.5%)
Duodenum tumors	6	4	2	54 (37–64)	6 (100.0%)
Reproductive cell tumors	6	3	3	34.5 (27–39)	6 (100.0%)
Cancers of multiple primary	92	58	34	63 (25–83)	87 (94.6%)
Cancers of unknown primary	11	8	3	66 (46–79)	11 (100.0%)
Undetermined pulmonary nodules	106	53	53	57 (24–87)	94 (88.7%)
Precancerous lesions	20	9	11	62 (43–81)	18 (90.0%)
Benign lesions	111	52	59	58 (21–81)	104 (93.7%)
Infectious diseases	52	32	20	58 (17–91)	51 (98.1%)
Total	2,360	1,267	1,093	59 (10–94)	2157 (91.4%)

CTC, circulating tumor cell.

### Comprehensive Atlas of Phenotypic and Karyotypic Characterization of CRCs

**Figure 2:**
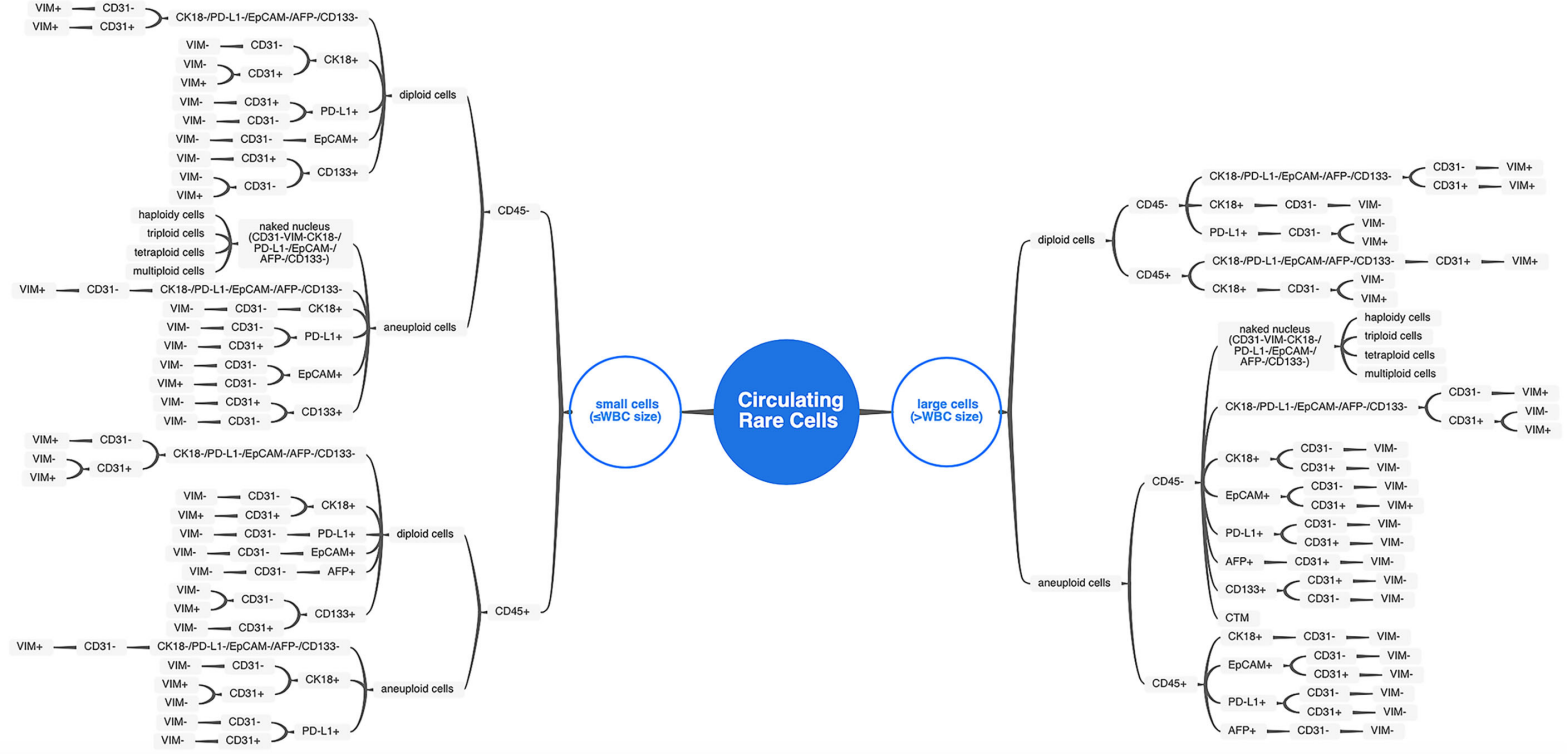
The comprehensive atlas categorizing CRC subtypes. Diverse CRC subtypes were categorized by cell size, biomarker expression, and chromosome 8 ploidy. For small cells (≤WBC size), distinguishing from WBC by hemocyte marker (CD45) took priority over all other features. For large cells (>WBC size), the chromosome 8 ploidy characterization comes to the first of subtypes classification.

**Figure 3:**
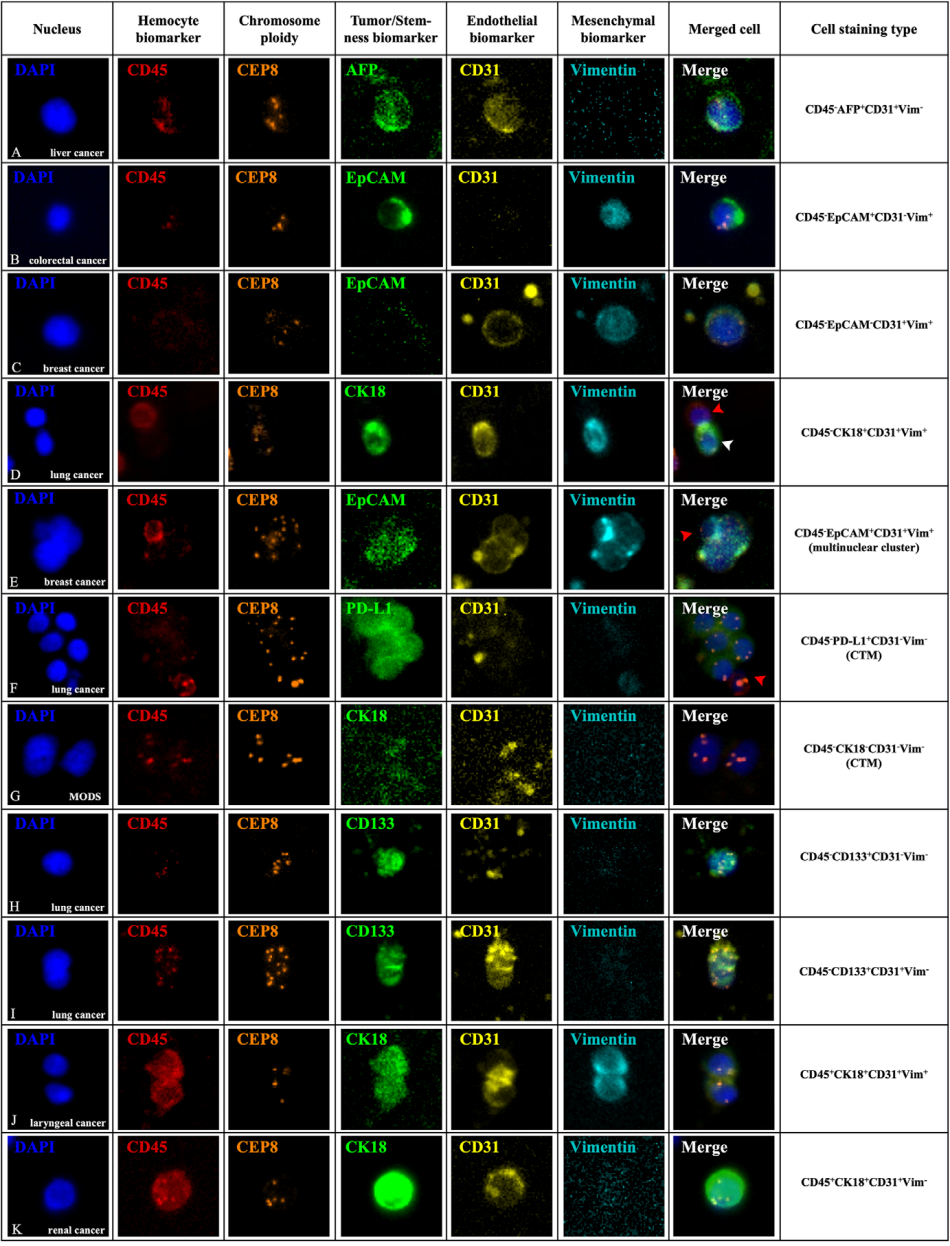
*In situ* phenotypic and karyotypic characterization of CRC subtypes in varieties of patients by SE-iFISH and image scanning platform. CRCs in peripheral blood were enriched and identified by 6-channel iFISH to co-characterize aneuploidy of chromosome 8 and a series of biomarker expression including hemocyte, tumor, and endothelial and mesenchymal markers. **(A)** An aneuploid hepatocellular carcinoma CTEC revealed a granule-like staining of secretory α-fetoprotein (AFP) from a liver cancer patient with local recurrence before interventional chemotherapy. **(B)** An aneuploid small colorectal cancer CTC had dual phenotypes of both epithelium (EpCAM) and mesenchyme (Vimentin) from a colorectal cancer patient during the tumor progression period after postoperative chemotherapy. **(C)** An aneuploid mesenchymal CTEC (CD45^-^EpCAM^-^CD31^+^Vim^+^) in a breast cancer patient during neoadjuvant endocrine therapy before surgery showing a strong intracellular distribution of CD31 and nuclear localization of Vimentin. **(D)** A diploid CRC in a lung cancer patient sampled after radical radiation therapy reveals positive expression of CK18, CD31, and Vimentin simultaneously (white arrow). An adjacent WBC (CD45^+^) is indicated by a red arrow. **(E)** A large aneuploid mesenchymal endothelial-epithelial fusion cluster (CD45^-^EpCAM^+^CD31^+^Vim^+^) in a breast cancer patient sampled when distant metastasis was detected after postoperative chemotherapy displayed a scattered vesicle-like staining of EpCAM in the nuclei and a positive staining of both CD31 and Vimentin. An attached mesenchymal WBC (CD45^+^Vim^+^) is indicated by a red arrow. **(F)** A CTM in a lung cancer patient with systemic multiple metastasis after 4 routine chemotherapy containing several aneuploid CTCs with nuclear localization of PD-L1. The adjacent WBC (CD45^+^PD-L1^-^) is indicated by a red arrow. **(G)** A null CTM from a non-neoplastic infectious patient had no biomarker expression. **(H)** An enriched aneuploid circulating stem-like cell displayed a strong expression of CD133 from a lung cancer patient sampled before radical surgery. **(I)** An endogenous cluster containing two aneuploid CTECs co-expressed CD133, showing CD45-/CD133+/CD31+/Vim- phenotype from peripheral blood in a lung cancer patient before radical surgery. **(J)** A CD45^+^ cell cluster consisting of two haploid cells with CK18, CD31, and Vimentin expression simultaneously enriched from a laryngeal cancer patient upon completion of radical surgery. **(K)** A large aneuploid CD45^+^ endothelial cell showed a strong expression of nuclear located CK18 in a renal cancer patient sampled when systemic multiple metastasis was detected after radical surgery. Fluorescence dyes conjugated to diverse antibodies (Cytelligen, USA): Alexa Fluor 488 (green), AF594 (red), Cyanine 5 (yellow), and Cy7 (pearl blue). Scale bar, 5 μm.

Apart from the existence of conventional CTCs and CTECs characterized by the criteria mentioned above, diverse circulating rare cells, including cells with size variation and heterogeneous expression of surface anchor molecules, were also enriched and comprehensively identified by the SE-iFISH and image scanning platform in varieties of cancer and non-neoplastic patients. A total of 71 subtypes of CRCs were classified according to cell size taking the average diameter of WBCs as the boundary, phenotyping of multiple cellular protein expression, and karyotyping of chromosome 8 ploidy (**Figure 2**). The representative images of multiple biomarkers distributed in CRCs with different morphological types are shown in **Figure 3**. Tumor biomarkers (CK18/PD-L1/EpCAM/AFP) and endothelial cell biomarker CD31 are heterogeneously localized in the cytoplasm, on the nuclear envelope, or in the nuclei, while stem cell biomarker CD133 and mesenchymal cell biomarker Vimentin mainly showed an intracellular distribution.

In contrast to typical CTCs and CTECs, a large amount of CRC subtypes have tumor biomarkers, CD31 and Vimentin expressed in pair with abnormal multiple chromosomes (**Figures 3A–C**); another subtype of diploid CRC with CK18/CD31/Vimentin-positive staining simultaneously was also observed in this study (**Figure 3D**). Examination of aneuploid multinuclear clusters enriched from a breast cancer patient demonstrated a distinct CRC subtype with the unique phenotype of CD45^-^/EpCAM^+^/CD31^+^/Vimentin^+^ (**Figure 3E**), indicating the existence of rarely reported “aneuploid mesenchymal epithelial-endothelial fusion cluster” in cancer patients. Besides individual CRCs, CTM which displayed CD45^-^/PD-L1^+^/CD31^-^/Vimentin^-^ phenotypes was also detected in diagnosed cancer patients’ peripheral blood (**Figure 3F**), and for the non-neoplastic infectious patients, we could only observe CTM with no biomarker expression (**Figure 3G**). Given the phenotypic and karyotypic heterogeneity, it would be significant to reveal different EMT or EndoMT statuses of carcinoma cells in the blood-borne dissemination or implantation procedure and further understand how CRC subtypes interplay in the peripheral circulation.

Another set of tri-marker (CD133/CD31/Vimentin)-iFISH which focused on the progenitor feature of the tumor demonstrated that cancer stem-like cells displayed a CD45^-^/CD31^-^/CD133^+^/Vimentin^-^ phenotype (**Figure 3H**). In addition, endogenous aneuploid cell co-expressed CD133, identified as the early circulating endothelial progenitor cells (CD31^+^/CD133^+^), were also detected (**Figure 3I**), showing the different phenotypic characterization of endothelial lineage differentiation together with the matured CD31^+^/CD133^-^ conventional CTECs.

Except for these CD45- non-hematopoietic cells and a small amount of CD45^+^ diploid cells which may be classified as remaining normal WBCs, we unexpectedly observed some CD45^+^ cell clusters and aneuploid cells expressing single or multiple biomarkers in both cancer and non-neoplastic patients. A representative cell cluster consisting of two haploid cells with CK18, CD31, and Vimentin in different expression patterns and a large endothelial cell showing a strong expression of CK18 with trisomy 8 is illustrated in **Figures 3J, K**, respectively; both were positive for CD45 staining. This suggested that besides possible residual WBCs, some distinct CD45^+^ cells might also provide one point of diverse CRC differentiation resource.

### Analysis of Total CTC and CTEC Numbers Among Different Disease Types

**Figure 4:**
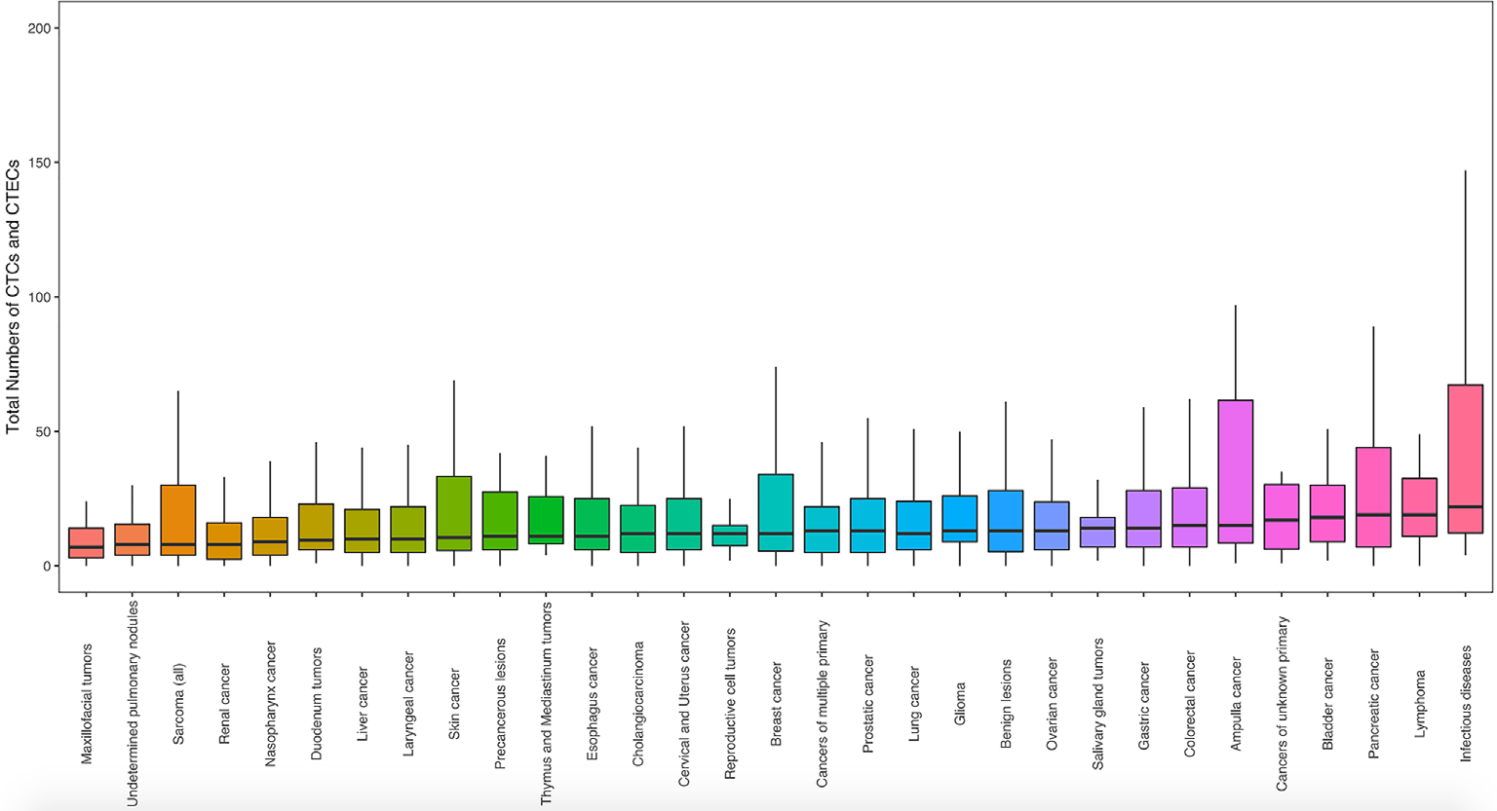
The landscape of total numbers of CTCs and CTECs identified for each disease type. CTC and CTEC counts were obtained using blood samples from patients in 31 groups of cancer and non-neoplastic diseases (n = 2,360). Statistical analysis was performed with the Kruskal–Wallis *H*-test. Box boundaries indicate the interquartile range; center lines: medians; whiskers: values within 1.5 interquartile ranges of the median.

The extending application of SE-iFISH and image scanning platform to co-detect CTCs and CTECs from 31 clinical disease types enabled us to make preliminary research on different disease types, among which CTCs and CTECs contained a wide range of total numbers, from 0 to 1,636 (**Figure 4** and **Table S4**). The median number of total CTCs&CTECs varied considerably by disease types, from a minimum of 7.00, for maxillofacial tumors, to a maximum of 22.00, for non-neoplastic infectious diseases. The relatively higher median number of CTCs&CTECs occurred in cancer types that are defined as digestive system neoplasm: pancreas (19.00), stomach (14.00), and ampulla and colorectum (15.00). The overall numbers of CTCs&CTECs among different disease types were statistically significant (p < 0.05). Notably, we observed a higher occurrence of CTCs and CTECs in non-neoplastic infectious diseases (22.00) than tumor disorders, including severe pneumonia, severe acute pancreatitis, acute peritonitis, infective endocarditis, gangrene, septic shock, and vascular trauma (**Table S3**), indicating highly heterogeneous of abnormal cells and the trends in the uncertainty of cancer classification simply through tumor biomarkers and endothelial cell biomarkers.

### Co-Detection of CEP8 and CEP12 and the Correlation Between Two Probes

**Figure 5:**
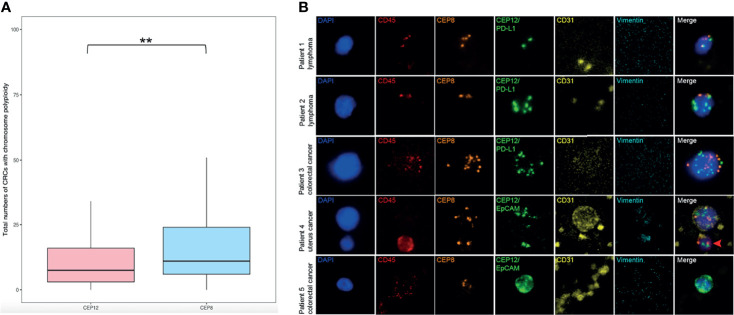
Characterization of double-probe (CEP8 and CEP12) co-detection of aneuploid CRCs. **(A)** Total numbers of CTCs and CTECs with chromosome polyploidy co-detected by CEP8 and CEP12 in a cohort of varieties of patients (n = 346). Statistical analysis was performed with a two-tailed *t-test*; **p < 0.01. **(B)** *In situ* phenotypic and karyotypic characterization of aneuploid CTCs and CTECs through a double-probe (CEP8 and CEP12) co-detection method. Ploidy combination of different patterns of two chromosomes (Chr) was visualized and described as aneuploidy Chr 8 and diploidy Chr 12 (Patient 1), diploid Chr 8 and aneuploidy Chr 12 (Patient 2), multiploidy Chr 8 and Chr 12 (Patient 3), aneuploidy Chr 8 and Chr 12 with CD31^+^ (Patient 4), and aneuploidy Chr 8 and Chr 12 with EpCAM^+^ (Patient 5); the WBC (CD45^+^) is indicated by a red arrow. Scale bar, 5 μm.

To detect the larger spectrum of highly heterogeneous CRCs, our study made a further trial using double chromosome enumeration probes, CEP8 and CEP12, in the process of immunofluorescence *in situ* hybridization for co-detection. We randomly collected 346 of all enrolled patients in this trial, including 305 patients with various cancers and 41 individuals with other non-neoplastic diseases (**Table S5**). Of all enriched cells with two-probe co-detection results, CEP8 and CEP12 shared a different-probe locus in the cell nucleus, showing that it would be precise to distinguish two ploidy patterns in the SE-iFISH Metafer platform. The total numbers of CRCs with chromosome 8 (Chr 8) polyploidy were higher than those of chromosome 12 (Chr 12) polyploidy (**Figure 5A**; CEP8 median = 12.0, CEP12 median = 8.0, *p* < 0.01), proving that CEP8 was the more significantly effective probe to identify CRCs with cytogenetic abnormalities of chromosome aneuploidy. However, we also observed the existence of generally considered normal cells (diploid Chr 8) with aneuploid Chr 12 in up to 57.27% of patients’ peripheral blood, and some of the non-hematologic CRCs (aneuploid Chr 12) had a different surface cellular protein expression as well (**Figure 5B**). Therefore, co-detection of CEP8 and CEP12 may ensure high specificity in detecting various CRCs performed by iFISH.

## Discussion

Given the hypothesis that any cell type eventually finds its way into the bloodstream deliberately or accidentally under pathological conditions (31), the spectrum of CRC may extend beyond its current boundary. Consequently, conventional detection methods that mainly target at CTCs, relying on either cell size or positive expression of a particular anchor protein on the cancer cell surface, are significantly limited. In our study, we applied an innovative detection technology integrating subtraction enrichment, immunostaining-fluorescence *in situ* hybridization (SE-iFISH), and a high-throughput automated image scanning system, independent of cell size variation and free of the unique molecule to effectively enrich and enable phenotyping multi-protein expression and karyotyping chromosome aneuploidy in comprehensive identification of CRCs. Our results revealed that the overall positive rate of CTC detection was 91.4% among varieties of patients, which is higher than the detection sensitivity documented by current conventional technologies that are restricted to EpCAM and/or CK expression (32). The positive incidences of CTCs in particular cancer types with a relatively large cohort of enrolled patients, such as 93.8% in lung cancer, 95.2% in colorectal cancer, 91.0% in cervical and uterus cancer, 92.7% in gastric cancer, 88.8% in liver cancer, and 91.3% in breast cancer, were in consistence with other negative enrichment methods (33, 34).

Our study expanded from the previous four-color mono-marker iFISH to the current six-color tri-marker iFISH (26), to allow the detection of epithelial, endothelial, mesenchymal, and stemness biomarkers in any triple combinations as well as aneuploid chromosomes at a time, making it possible to discover cells in non-reported phenotypes and investigate how different biomarkers interplay on highly heterogeneous CRCs. With the novel SE-iFISH and image scanning platform, we mapped out an atlas of 71 kinds of CRC subtypes for the first time, outlining the phenotypic and karyotypic characterization based on the investigation of a large cohort of 2,360 patients.

The existence of single aneuploid CTCs or CTECs has been widely discussed in clinical studies with the application of various detection technologies (35, 36). Of note is the insight about the potential differentiation capability of CRCs in peripheral blood and possible EMT/EndoMT process whereby single cells and cell clusters displayed cell size variation and distinct phenotypes, which may lend color to the works that we detected a wide range of CRC subpopulations. Among the various CRC phenotypes observed, the presence of epithelial marker expression is of low-frequency detection, whereas aneuploidy cells with endothelial features were very prevalent. It is believed that tumor cells must possess the capacity to form the tumor microenvironment of metastasis with perivascular macrophages and endothelial cells for hematogenous dissemination (37), while the intravasation process is probably not accessible for all initial phenotypes of cells (38). This may explain the fact that those epithelial marker-positive subtypes in primary tumors were usually absent among CRCs and the presence of cytogenetic abnormality of endothelial cells. Besides, it has been implied that small mesenchymal CRCs may be more closely related to EMT or EndoMT and responsible for cancer relapse (34, 39). Most of the Vimentin^+^ aneuploidy CRCs observed in the current work were smaller in size than other cells, which was in accordance with previous reports (40, 41). Furthermore, the rarely reported aneuploid mesenchymal (Vimentin^+^) CTC-CTEC fusion cluster expressing both the epithelial marker EpCAM and the endothelial marker CD31, as well as large CTM with a series biomarkers expressing was also demonstrated in the present study, showing a significant interaction of tumor cells (TCs), endothelial cells (TECs), and mesenchymal cells (TMCs) during the process of EMT/EndoMT in peripheral circulation or implantation of cancer cells (30, 42). CD133 is a stemness marker that is related to increased tumor-initiating ability, tumor progression, and cancer recurrence in numerous types of cancer (43); thus, the CD133 expression cell subset is generally referred to as a colony-forming cell that possesses seed-cell property and provides an explanation of self-renewal and metastatic potential of cancer cells (31, 44, 45). Previously, mature CRCs were distinguished from progenitor cells based on CD133 expression. Our results addressed aneuploidy cells with stem and EndoMT features that exist simultaneously in the circulation of carcinoma patients, indicating that the EndoMT process may lead to the acquisition of a stem cell-like phenotype by CRCs (46), which is attributed to CRC variants during tumor metastasis. The presence of EMT or EndoMT and the progenitor properties of CRCs may come to the most important among the many heterogeneity characteristics in cancer diseases, determining cell resistance to apoptosis, invasiveness, and survival ability to the immunological surveillance (47), and subsequently play a critical role in tumor metastasis and disease prognosis. There is, however, no consensus by far on those particular CRC phenotypes, thus contributing to cancer progression, invasion, and metastasis. In this regard, assessment of more possible biomarker co-expression, analysis of the correlation between specific phenotypes and cancer stages, and adequate approaches for molecular profiling, including both protein and genomic profiling performed on a single CRC, are necessary for further investigation.

Most CRCs that have been substantially discussed in extensive studies are tumor-derived and of non-hematologic character (CD45^-^). Interestingly, we observed an amount of CD45^+^ aneuploid cells and clusters expressing single or multiple biomarkers when analyzing the scanned images. Previous studies have demonstrated that the most representative populations of hematologic CRCs were tumor cells of lymphoma and myeloma (36). Moreover, in the realization of the awareness that the majority of CRCs may be derived from the stem cell or hemangioblast that possesses multi-lineage differentiation potential (31), CD45^+^ phenotypes of CRCs may be considered as a colony of cells that are at different stages of differentiation from progenitor type to functional mature type (26), which were distinguishable based on their proliferative capacity. Concerning more CRC types than herein identified, a clinical interpretation of CRC entails definitions of more diverse biomarkers especially other hematopoietic or progenitor cell markers such as AC133, CD14, and CD34, as well as the establishment of robust specificity cutoffs for quantitatively analysis of CRCs on varieties of patients (48).

Beyond progenitor cells and EMT/EndoMT-related phenotype shifting of CRCs, there is a large amount of CRCs at the “naked nuclei” status without any biomarker expression described in this study, which also requires comprehensive identification and characterization. The population of “naked nuclei” cells in circulation has features similar to those of rare megakaryocytes, but usually with more evident atypia manifesting as irregular nuclear profiles and aneuploid chromosomes. It is assumed that the origin of “naked nuclei” is the malignant cells from which the outer membrane of the nuclear envelope was partially detached mechanically during the process of intravasation and extravasation. However, with the development of the CRC detection strategy, the lack of a reliable nuclear or cytoplasm antigen marker for those cells is believed to impair definitive conclusions (49). Regarding this, the developed SE-iFISH and image scanning platform allows for any new biomarkers to effectively identify specific disease-associated CRCs.

A subgroup analysis among varieties of diseases revealed a comparatively high amount of CTCs and CTECs in non-neoplastic infectious individuals and patients with benign lesions. Cases with the presence of some non-clustered mononuclear polyploid abnormal CRCs were also occasionally detected, whereas multinuclear aneuploid CRC clusters with expression of a series of biomarkers were observed only in cancer patients. Since they are exposed to different external factors in physiological conditions, human somatic tissues can contain a small fraction of aneuploid cells at a lower grade of pathological potential (50, 51); we suspected that an ordinary inflammatory response caused by acute infectious and benign occupying lesions may amplify this chromosomal abnormality and have a close association with induced aneuploidy (52) and therefore increase the positive rates of CTC in peripheral blood. Additionally, it has been reported that aberrant endothelial cell elevation occurs in either vascular injury-related inflammation or tumor neo-vascularization (53), and mature endothelial cells and endothelial progenitors may interact with each other in inflammation where mature cells were activated and the progenitor’s dysfunction was induced for the subsequent increase of CTEC (31, 54). Still, the clear reason for these results remains to be further studied, while the difference between the CTCs detected in non-neoplastic diseases and those cancer-related CTCs, whether these CTCs were correlated with the possible cancerous process of some benign lesions, and exploring of more CRC subtypes expressing specific biomarkers in certain disease are also worth intensive research. This is exactly the important reason that we introduced the concept of CRC to increase diagnostic specificity in clinical practice by a greater diversity of possible biomarkers and chromosomal abnormalities. We hope that our study can avoid a serious pitfall when using CTCs/CTECs as independent markers in cancer diagnosis and open avenues for learning how to navigate these complexities.

Another extraordinary finding of this study was a higher specificity and efficiency of CRC detection by the double-probe (CEP8 and CEP12) method. Centromere probe 8 was the conventionally used FISH probe approved by the USFDA to identify aneuploid solid tumor cells (30). However, analysis of double probe co-detection in this preliminary study performed on a portion of this cohort showed that 57.27% of patients with normal diploid Chr 8 possessed aneuploid Chr 12, suggesting a relatively high false-negative CRC detection through a single probe. Since abnormal ploidy of specific chromosomes in neoplastic cells impacts on transcription of multiple genes (55), resulting in a profound variety of phenotypes that subsequently contribute to tumor heterogeneity, cancer relapse (56), and therapeutic outcome (57), the karyotypic characterization of Chr 12, as well as the combination of Chr 8 and Chr 12 in varieties of patients, remained to be further investigated.

In summary, based on detection of the results of 3,476 peripheral blood samples from a cohort of 2,360 cancer and non-neoplastic patients, the present study first-ever mapped out an atlas categorizing the diverse CRCs into 71 subtypes by cell size, biomarker expression, and chromosome ploidy and therefrom conducted a preliminary analysis of CRC subpopulations among grouped disease types. However, the current SE-iFISH and image scanning platform with at most six colors of fluorescence channels may still have the biomarker co-detection limitation, biomarker and chromosome enumeration probe development for CRC detection is in its infancy, and it shall be noted that the rare cell types listed are not exhaustive by far. Future extensive investigation on the proliferation and differentiation of CRCs in either circulation or tumor microenvironment, what the clinical significance of CD45^+^ blood cells with aberrant phenotypic and karyotypic characterization, CTM or rare fusion clusters have, and establishment of standardization of cutoff thresholds for diagnosis or prognosis of specific diseases will shed light on a better understanding of the diverse categories of CRCs and subsequently benefit personalized clinical management for varieties of patients.

## Conclusion

This is the first cell-based liquid biopsy study to report the exhaustive subtypes of CRCs in a large clinical cohort with a comprehensive atlas outlining the phenotypic and karyotypic characterization of the diverse CRCs. The improved SE-iFISH and high-throughput automated image scanning platform, integrating with double-probe (CEP8 and CEP12) co-detection and a series of biomarkers immunostaining simultaneously, provides a unique strategy to effectively detect CRCs by targeting alternative biomarkers and chromosomes that are of particular interest in clinical research. We here make an elaborate description of the EMT or EndoMT phenomenon, the progenitor properties of abnormal cells, and the rarely reported aneuploidy blood cells expressing single or multiple biomarkers. A preliminary quantitative analysis suggested a high occurrence of CTCs and CTECs in non-neoplastic infectious diseases. Based on an entire spectrum of CRC acting and reacting as part of a specific disease, greater diagnostic potential, cancer metastasis monitoring, and personalized treatment benefits can be well worth expecting.

## Data Availability Statement

The original contributions presented in the study are included in the article/**Supplementary Material**. Further inquiries can be directed to the corresponding authors.

## Ethics Statement

The studies involving human participants were reviewed and approved by the Ethical Review Board of Shanghai General Hospital, Shanghai Jiao Tong University School of Medicine, China (2016KY130). Written informed consent to participate in this study was provided by the participants’ legal guardian/next of kin.

## Author Contributions

QH and JC designed the study. BH, YW, and JX performed the experiments. BH collected the clinical information. BH, JC, YG, YW, and JX analyzed the data. All authors participated in the discussion and interpretation of the results. BH wrote the manuscript. QH and JC contributed to the manuscript revision. All authors contributed to the article and approved the submitted version.

## Funding

This work was supported by a grant from the Shanghai “Rising Stars of Medical Talents” Youth Development Program (No. SHWRS (2021_099) to JC).

## Conflict of Interest

The authors declare that the research was conducted in the absence of any commercial or financial relationships that could be construed as a potential conflict of interest.

## Publisher’s Note

All claims expressed in this article are solely those of the authors and do not necessarily represent those of their affiliated organizations, or those of the publisher, the editors and the reviewers. Any product that may be evaluated in this article, or claim that may be made by its manufacturer, is not guaranteed or endorsed by the publisher.
