# Supplementary material for: Comprehensive Atlas of Circulating Rare Cells Detected by SE-iFISH and Image Scanning Platform in Patients With Various Diseases
Source: Front Oncol. 2022 Mar 2;12:821454. doi: 10.3389/fonc.2022.821454 (PMC8924462; doi:10.3389/fonc.2022.821454)
Supplement: Supplementary file 1 [file Table_1.docx]

**Table S1 the prevalence of CTC&CTECs identified for each specific disease in precancerous lesions group**

| **Disease Type** | **Patients number, *n*** | **CTC&CTECs, *median*** |
| --- | --- | --- |
| colon villous tubular adenoma with epithelial dysplasia | 5 | 9.0 |
| pulmonary atypical adenomatous hyperplasia | 4 | 5.5 |
| cervical high-grade intraepithelial neoplasia（CINIII） | 3 | 6.0 |
| rectal villioustublar adenoma | 3 | 53.5 |
| gastric intraepithelial neoplasia | 2 | 36.5 |
| atypical hyperplasia of endometrium | 1 | 8.0 |
| duodenal epithelial dysplasia | 1 | 14.5 |
| appendix sessile serrated adenoma (SSA) | 1 | 12.0 |

**Table S2 the prevalence of CTC&CTECs identified for each specific disease in benign lesions group**

| **Disease Type** | **Patients number, *n*** | **CTC&CTECs, *median*** |
| --- | --- | --- |
| cryptogenic organizing pneumonia (COP) | 20 | 9.0 |
| pulmonary granulomatous inflammation | 14 | 11.0 |
| pulmonary nodules (lymphoid hyperplasia) | 13 | 18.0 |
| mediastinal cyst | 8 | 6.5 |
| pulmonary hamartoma | 5 | 8.0 |
| ovarian cyst | 5 | 36.0 |
| pulmonary tuberculosis | 4 | 16.5 |
| gastric ulcer | 4 | 35.5 |
| common bile duct calculus | 3 | 31.0 |
| bile duct stenosis (no malignant change) | 3 | 21.5 |
| hysteromyoma | 3 | 55.0 |
| internal carotid aneurysm | 3 | 6.0 |
| crohn‘s disease | 2 | 73.5 |
| cerebral hemorrhage | 2 | 93.5 |
| necrotizing intestinal obstruction | 1 | 710.0 |
| hyperplastic intestinal obstruction | 1 | 9.0 |
| pulmonary lipomyoma | 1 | 0.0 |
| lung hernia | 1 | 5.0 |
| spontaneous pneumothorax | 1 | 4.0 |
| sclerosing pneumocytoma | 1 | 4.0 |
| lacteal cyst | 1 | 2.0 |
| breast fibroadenoma | 1 | 0.0 |
| (chest) heterotopic liver | 1 | 4.0 |
| esophageal lymphohyperplasia | 1 | 40.0 |
| gastric polyposis | 1 | 10.0 |
| Brunner gland adenoma | 1 | 90.0 |
| hyperplastic proctitis | 1 | 129.0 |
| retroperitoneal schwannoma | 1 | 2.0 |
| liver transplanted condition (cirrhosis) | 1 | 11.0 |
| ovarian mucinous cystadenoma | 1 | 22.5 |
| ovarian fibroma | 1 | 28.0 |
| parotid Warthin tumor | 1 | 9.0 |
| nasal polyposis | 1 | 38.0 |
| pituitary adenoma | 1 | 27.0 |
| Langerhans cell granuloma disease | 1 | 93.5 |
| lymphnoditis | 1 | 4.0 |

**Table S3 the prevalence of CTC&CTECs identified for each specific disease in non-neoplastic infectious diseases group**

| **Disease Type** | **Patients number, *n*** | **CTC&CTECs, *median*** |
| --- | --- | --- |
| severe pneumonia | 13 | 25.0 |
| severe acute pancreatitis | 10 | 15.0 |
| cholangitis | 7 | 19.0 |
| acute diffuse peritonitis | 6 | 75.0 |
| septic shock | 5 | 30.0 |
| hepatapostema | 3 | 25.0 |
| multiple organ dysfunction syndrome (MODS) | 2 | 15.0 |
| septicopyemia | 2 | 19.5 |
| severe myocarditis | 1 | 65.0 |
| gangrene (DIC) | 1 | 22.0 |
| infective endocarditis | 1 | 12.0 |
| pulmonary abscess | 1 | 6.0 |

**Table S4. Statistical characteristics of total numbers of CTCs and CTECs identified for each disease type**

| **Disease Type** | **median** | **lower quantiles** | **upper quantiles** |  |
| --- | --- | --- | --- | --- |
|  |  |  |  |  |
| Maxillofacial tumors | 7.00 | 3.00 | 14.00 |  |
| Undetermined pulmonary nodules | 8.00 | 4.00 | 15.50 |  |
| Sarcoma (all) | 8.00 | 4.00 | 30.00 |  |
| Renal cancer | 8.50 | 2.75 | 20.00 |  |
| Nasopharynx cancer | 9.00 | 4.00 | 18.00 |  |
| Duodenum tumors | 9.50 | 6.00 | 23.00 |  |
| Liver cancer | 10.00 | 5.00 | 21.50 |  |
| Laryngeal cancer | 10.50 | 5.00 | 22.00 |  |
| Skin cancer | 10.50 | 5.75 | 33.75 |  |
| Precancerous lesions | 11.00 | 6.00 | 27.50 |  |
| Thymus and Mediastinum tumors | 11.00 | 8.50 | 33.50 |  |
| Esophagus cancer | 11.50 | 6.00 | 25.75 |  |
| Cholangiocarcinoma | 12.00 | 5.00 | 22.50 |  |
| Cervical and Uterus cancer | 12.00 | 6.00 | 15.00 |  |
| Reproductive cell tumors | 12.00 | 7.50 | 15.00 |  |
| Breast cancer | 12.50 | 6.00 | 35.00 |  |
| Cancers of multiple primary | 13.00 | 5.00 | 22.00 |  |
| Prostatic cancer | 13.00 | 5.00 | 25.00 |  |
| Lung cancer | 13.00 | 6.00 | 24.25 |  |
| Glioma | 13.00 | 9.00 | 26.00 |  |
| Benign lesions | 13.50 | 5.75 | 31.25 |  |
| Ovarian cancer | 13.50 | 6.00 | 25.00 |  |
| Salivary gland tumors | 14.00 | 7.00 | 18.00 |  |
| Gastric cancer | 14.00 | 7.00 | 30.50 |  |
| Colorectal cancer | 15.00 | 7.00 | 29.00 |  |
| Ampulla cancer | 15.00 | 8.50 | 61.50 |  |
| Cancers of unknown primary | 17.00 | 6.25 | 30.25 |  |
| Bladder cancer | 18.00 | 9.00 | 30.00 |  |
| Pancreatic cancer | 19.00 | 7.00 | 44.00 |  |
| Lymphoma | 19.00 | 11.00 | 32.50 |  |
| Infectious diseases | 22.00 | 12.75 | 74.00 |  |

**Table S5. Clinical characteristics of patients enrolled in co-detection of CEP8&CEP12 trial (n=346)**

| **Disease Type** | **Total number, *n*** | **Gender, *n(%)*** | | **Age, *median(range)*** |
| --- | --- | --- | --- | --- |
|  |  | **Male** | **Female** |  |
| Lung cancer | 18 | 14 | 4 | 66.5 (37-79) |
| Colorectal cancer | 83 | 50 | 33 | 63 (30-82) |
| Cervical and Uterus cancer | 47 | 0 | 47 | 54 (27-85) |
| Gastric cancer | 50 | 33 | 17 | 64.5 (35-84) |
| Nasopharynx cancer | 11 | 8 | 3 | 55 (36-79) |
| Liver cancer | 5 | 3 | 2 | 56 (36-64) |
| Breast cancer | 8 | 0 | 8 | 62.5 (47-80) |
| Laryngeal and Thyroid cancer | 9 | 8 | 1 | 63 (61-76) |
| Pancreatic cancer | 15 | 5 | 10 | 64 (46-75) |
| Esophagus cancer | 3 | 2 | 1 | 61 (38-82) |
| Ovarian cancer | 20 | 0 | 20 | 57 (32-90) |
| Cholangiocarcinoma | 3 | 1 | 2 | 62 (59-65) |
| Lymphoma | 3 | 2 | 1 | 71 (58-72) |
| Parotid cancer | 3 | 1 | 2 | 71 (29-72) |
| Ampulla cancer | 4 | 1 | 3 | 66.5 (56-75) |
| Other cancers | 6 | 5 | 1 | 64.5 (43-68) |
| Cancers of multiple primary | 13 | 9 | 4 | 65 (42-79) |
| Cancers of unknown primary | 3 | 1 | 2 | 69 (47-70) |
| Precancerous lesions | 7 | 4 | 3 | 51 (43-81) |
| Benign lesions | 12 | 5 | 7 | 54 (31-68) |
| Infectious diseases | 23 | 12 | 11 | 63 (24-77) |
| Total | 346 | 164 | 182 | 62 (24-90) |
